# Obesity, Sarcopenia and Myosteatosis: Impact on Clinical Outcomes in the Operative Management of Crohn’s Disease

**DOI:** 10.1093/ibd/izad225

**Published:** 2023-10-20

**Authors:** Mark Donnelly, Dorothee Driever, Éanna J Ryan, Jessie A Elliott, John Finnegan, Deirdre McNamara, Ian Murphy, Kevin C Conlon, Paul C Neary, Dara O Kavanagh, James M O’Riordan

**Affiliations:** Department of Surgery, Tallaght University Hospital, Dublin, Ireland; Department of Surgery, Tallaght University Hospital, Dublin, Ireland; Department of Surgery, Tallaght University Hospital, Dublin, Ireland; Department of Surgery, Tallaght University Hospital, Dublin, Ireland; Department of Radiology, Tallaght University Hospital, Dublin, Ireland; Department of Gastroenterology, Tallaght University Hospital, Dublin, Ireland; School of Medicine, Trinity College Dublin, The University of Dublin, DublinIreland; Department of Radiology, Tallaght University Hospital, Dublin, Ireland; Department of Surgery, Tallaght University Hospital, Dublin, Ireland; School of Medicine, Trinity College Dublin, The University of Dublin, DublinIreland; Department of Surgery, Tallaght University Hospital, Dublin, Ireland; School of Medicine, Trinity College Dublin, The University of Dublin, DublinIreland; Department of Surgery, Tallaght University Hospital, Dublin, Ireland; Royal College of Surgeons in Ireland, Department of Surgical Affairs, Dublin, Ireland; Department of Surgery, Tallaght University Hospital, Dublin, Ireland; School of Medicine, Trinity College Dublin, The University of Dublin, DublinIreland

**Keywords:** inflammatory bowel disease, Crohn’s disease, body composition, lean body mass, fat mass, sarcopenia, nutrition, computed tomography, visceral fat, subcutaneous fat, obesity, comprehensive complications index

## Abstract

**Background:**

Obesity, sarcopenia, and myosteatosis in inflammatory bowel disease may confer negative outcomes, but their prevalence and impact among patients with Crohn’s disease (CD) have not been systematically studied. The aim of this study was to assess nutritional status and body composition among patients undergoing resectional surgery for CD and determine impact on operative outcomes.

**Methods:**

Consecutive patients with CD undergoing resection from 2000 to 2018 were studied. Total, subcutaneous, and visceral fat areas and lean tissue area (LTA) and intramuscular adipose tissue (IMAT) were determined preoperatively by computed tomography at L3 using SliceOmatic (Tomovision, Canada). Univariable and multivariable linear, logistic, and Cox proportional hazards regression were performed.

**Results:**

One hundred twenty-four consecutive patients were studied (ileocolonic disease 53%, *n* = 62, biologic therapy 34.4% *n* = 43). Mean fat mass was 22.7 kg, with visceral obesity evident in 23.9% (*n* = 27). Increased fat stores were associated with reduced risk of emergency presentation but increased corticosteroid use (β 9.09, standard error 3.49; *P* = .011). Mean LBM was 9.9 kg. Sarcopenia and myosteatosis were associated with impaired baseline nutritional markers. Myosteatosis markers IMAT (*P* = .002) and muscle attenuation (*P* = .0003) were associated with increased grade of complication. On multivariable analysis, IMAT was independently associated with increased postoperative morbidity (odds ratio [OR], 1.08; 95% confidence interval (CI), 1.01-1.16; *P* = .037) and comprehensive complications index (*P* = .029). Measures of adiposity were not associated with overall morbidity; however, increased visceral fat area independently predicted venous thromboembolism (OR, 1.02; 95% CI, 1.00-1.05; *P* = .028), and TFA was associated with increased wound infection (OR, 1.00; 95% CI, 1.00-1.01; *P* = .042) on multivariable analysis.

**Conclusion:**

Myosteatosis is associated with nutritional impairment and predicts increased overall postoperative morbidity following resection for CD. Despite its association with specific increased postoperative risks, increased adiposity does not increase overall morbidity, reflecting preservation of nutritional status and relatively more quiescent disease phenotype. Impaired muscle mass and function represent an appealing target for patient optimization to improve outcomes in the surgical management of CD.

**Key Messages**

**What is already known?**

Obesity, sarcopenia, and myosteatosis are common in inflammatory bowel disease. Their prevalence and impact in CD patients undergoing operative intervention are incompletely understood.

**What is new here?**

Myosteatosis was independently associated with postoperative morbidity in patients undergoing resectional surgery for CD. Sarcopenia in the present study appeared to play a lesser role in surgical outcomes; however, it is still prevalent within CD patients and may offer useful insight into the overall preoperative body composition of patients at potential risk of postoperative complications.

**How can this study help patient care?**

This study provides new insights into assessment of patients with Crohn’s disease prior to surgical intervention. This information may be useful in both a prehabilitation and rehabilitation setting for CD patients undergoing surgery.

## Introduction

Inflammatory bowel disease (IBD) is a chronic inflammatory disorder of the gastrointestinal tract. The 2 main subtypes are Crohn’s disease (CD) and Ulcerative colitis (UC). The incidence of CD is 6.3 per 100 000 person years in Northern Europe.^1^ The pathophysiology of IBD is complex and multifactorial, consisting of complex interactions between environment, host immune response, and genetic susceptibility. Consequently, the management of CD involves a multidisciplinary approach between gastroenterology and surgery. The rate of CD patients requiring surgical intervention has declined over the last 2 decades, most likely associated with the advent of anti-TNF therapy.^2,3^ In the UK population, the risk of surgery for CD at 10 years has reduced from 44% to 21% over this time period, with similar rates of decline observed in a Swiss 10-year population based study.^4^ However, while there has been an improvement in the overall management of IBD, there is an accepted progression towards a more complicated disease phenotype in patients who do not respond favorably or stop responding to medical therapy, with both emergency and elective surgery being common.^5^ Crohn’s disease patients endure significant nutritional challenges associated with symptoms and acute/chronic inflammatory states resulting in malabsorption.

Postoperative complications in CD patients undergoing bowel resection remain at a high incidence, with reports ranging from 20% to 40%.^6,7^ Often reported causes of postoperative morbidity in CD patients is their preoperative clinical status: chronic malabsorptive state, acute/chronic inflammatory state, immunosuppression, intestinal obstruction secondary to structuring, and localized or systemic sepsis associated with disease flare.^8^ Consequently, the acute physiological stress and metabolic response to injury postoperatively in this group of patients is an important consideration that presents its own challenges. Optimal management and choice of surgical approach commonly require multiple investigations to ascertain the level of disease activity, severity, and anatomical site of concern. Computed tomography (CT) is the investigation of choice in this regard; however, it may also provide pertinent information relating to patients’ overall preoperative health, nutritional and body composition status, in particular adiposity, muscle mass, and quality.^9^

Sarcopenia has been defined as a low muscle mass with either decreased muscle strength and/or reduced physical performance.^10^ It is characterized by a reduction in skeletal muscle mass and function and may be a feature of malnutrition common in IBD. Sarcopenia is well-characterized as a predisposing factor contributing to adverse outcomes in several clinical contexts including postoperative morbidity.^11^ The natural history of sarcopenia is well-understood, with the drivers of disease multifactorial in nature. Reduced physical activity, systemic inflammation, reduced calorific intake, and increased metabolic rate are all contributors.^12^ Myosteatosis is defined as the process by which skeletal muscle becomes infiltrated with lipid into both the inter- and intramuscular compartments.^13^ It is recognized to negatively impact muscle mass, mobility, strength, and muscular metabolism. Both sarcopenia and myosteatosis have been shown to predispose both oncological and nononcological patients to adverse outcomes, increased morbidity, mortality, and reduced quality of life.^14-20^ Visceral obesity may be associated with the clinical phenotype of metabolic syndrome, with type 2 diabetes, hypertension and hyperlipidaemia, micro-albuminuria, endothelial dysfunction, and a pro-inflammatory and pro-thrombotic state, with increased levels of circulating C-reactive protein (CRP), tumor necrosis factor-α (TNF-α), and interleukin-6 (IL-6).^21,22^

While these risk factors are often present in patients with CD, few studies have assessed the prevalence and impact of obesity, sarcopenia, and myosteatosis among this group. Studies to date have been limited by the heterogeneity in treatment, reporting of body composition parameters, inconsistencies in nomenclature, and the use of nonstandard measurements that have not been validated in nutritional research.^23-28^ The present study therefore aims to characterize nutritional parameters and body composition among patients with CD and determine the impact of body composition on operative outcomes. We hypothesized that measures of increased adiposity, as well as reduced skeletal muscle mass and myosteatosis, would be associated with adverse outcomes among patients with CD undergoing operative management.

## Methods

### Patient Selection and Study Design

Tallaght University Hospital, Dublin, Ireland is a tertiary referral center for colorectal surgery and the management of IBD. Records for all patients diagnosed with CD treated with operative management between 2000 and 2018 were reviewed for inclusion. All patients with at least 1 preoperative CT scan capturing the level of the L3 vertebra conducted at our center and available for review were included for analysis of operative outcome. All emergency patients included in the analysis had a preoperative CT performed on the same admission. Patients without a preoperative CT were excluded from the study. One of the included patients had a diagnosis of rheumatoid arthritis; this was deemed to be well controlled clinically, in remission on preoperative imaging, and therefore noncontributory with regard to body composition. No patients with a history of eating disorder, actively malignancy, poorly controlled chronic rheumatic diseases, or other illness known to influence body composition were included in the final analysis. This study was granted ethical approval by the TUH Research Ethics Committee.

### Treatment Protocol

All elective patients within the study were reviewed at the IBD multidisciplinary team (MDT) meeting prior to being listed for surgery. Patients were classified according to the Montreal revision of the Vienna classification based on age at diagnosis, location, and behavior.^29,30^ Patients were assessed by an IBD clinical nurse specialist in the outpatient setting. Preoperative blood samples were taken as part of a nutritional assessment, assessment of anemia, and investigation of electrolyte imbalances. A malnutrition universal screening tool (MUST) was also used to screen each patient, as well as anthropometric measurements to assess preoperative nutritional status. Preoperative optimization for patients requiring surgical resection was routinely undertaken. A full ileocolonoscopy, usually with a preoperative CT scan +/- magnetic resonance enterogram (MRE) were performed in the outpatient setting for assessment of extent of disease, anatomical location of disease, active complications of disease, preoperative decision-making, and disease phenotyping. Preoperative assessment and anesthetic clinic were utilized where deemed necessary. Since 2007, enhanced recovery after surgery (ERAS) protocols were implemented, with laparoscopy the preferred operative approach where feasible.

Emergency presentations were managed as per institutional protocols for emergent surgical admissions, in keeping with evidence-based guidelines published by the European Crohn’s and Colitis Organization (ECCO) on the management of Crohn’s disease in the emergency setting.^31^

### Data Collection and Study Definitions

Data were collected from a prospectively maintained database and transferred into a standardized data collection spreadsheet. Collected data included demographics, comorbidities and performance status, predefined body composition parameters/nutritional parameters, pathologic characteristics, disease location and severity, previous treatment, operative details, postoperative morbidity, recurrence, and associated treatment data. Postoperative complications were coded using the Clavien-Dindo classification and the comprehensive complications index (CCI), with complications graded IIIa-IVb defined as major morbidities.^32,33^ The reported postoperative complications included reoperation, anastomotic leak, venous thromboembolism (VTE), wound infection, infective complications, and incisional hernia (Table 1). Postoperative complications were measured until 6 months after resectional surgery was performed.

**Table 1.** Clinicopathologic characteristics of study population.

Clinical Characteristics	
Age, years, mean (SD)	39.3 (15.5)
Sex, *n* (%)	
Female	67 (54)
Male	57 (46)
ASA grade, *n* (%)	
ASA1	90 (72.6)
ASA2	30 (24.2)
ASA3	4 (3.2)
Smoking status, *n* (%)	
Never	98 (79.0)
Former	19 (15.3)
Current	4 (3.2)
Pathologic characteristics	
Phenotype at surgery, *n* (%)	
Stricturing	52 (41.9)
Penetrating	27 (21.8)
Both	4 (3.1)
Site of disease at surgery, *n* (%)	
Terminal ileum	17 (13.7)
Colon	25 (20.1)
Ileocolonic	62 (50)
Perianal	2 (1.6)
Multifocal	6 (4.8)
Other	5 (4.0)
Extraintestinal manifestations, *n* (%)	
Arthritis (Rheamuatoid = 1, Psoriatic = 1, Enteropathic = 7)	9 (7.3)
Uveitis	2 (1.6)
Erythema nodosum	1 (0.8)
Pyoderma gangrenosum	5 (4.0)
Treatment characteristics	
Previous treatment, *n* (%)	
Medical therapy	95 (76.6)
Biologic therapy	43 (34.6)
Surgery	33 (26.6)
Preoperative corticosteroids, *n* (%)	31 (25)
Indication for surgery, *n* (%)	
Perforation	6 (4.8)
Stricture	42 (33.9)
Fistula	20 (16.1)
Refractory to medical therapy	26 (20.1)
Other	11 (8.9)
Surgical urgency, *n* (%)	
Planned	96 (77.4)
Emergent	8 (6.5)
Surgical procedure, *n* (%)	
Small bowel resection	8 (6.5)
Ileocolic resection	87 (70.2)
Other	30 (24.2)
Anastomosis, *n* (%)	70 (56.5)
Stoma, *n* (%)	18 (14.5)
Postoperative outcomes	
Clavien-Dindo grade, *n* (%)	
Grade I	76 (61.3)
Grade II	11 (8.9)
Grade IIIa	20 (16.1)
Grade IIIb	12 (9.7)
Grade IVa	3 (2.4)
Grade IVb	3 (2.4)
Grade V	0 (0.0)
Reoperation, *n* (%)	3 (2.4)
Leak, *n* (%)	7 (5.6)
Venous thromboembolism, *n* (%)	5 (4.0)
Wound infection, *n* (%)	12 (9.6)
Infective complications, *n* (%)	28 (22.6)
Incisional hernia, *n* (%)	5 (4.0)
Inpatient length of stay, median (range), days	13 (1–270)
Critical care length of stay, median (range), days	0 (0–18)
Comprehensive complications index, mean (SD)	9.49 (17.39)

Abbreviations: ASA, American Society of Anaesthetists; SD, standard deviation

**Table 2.** Body composition and nutritional assessment of study population.

Body Composition	
Visceral fat area, cm^2^, mean (SD)	82.4 (80.4)
Subcutaneous fat area, cm^2^, mean (SD)	190.8 (145.3)
Total fat area, cm^2^, mean (SD)	273.2 (194.3)
Fat mass, kg, mean (SD)	22.7 (8.2)
Visceral obesity, *n* (%)	27 (23.9)
Lean tissue area, cm^2^, mean (SD)	126.5 (35.9)
Intramuscular adipose tissue, cm^2^, mean (SD)	8.6 (9.0)
Muscle attenuation, HU, mean (SD)	38.7 (11.0)
Lean body mass, kg, mean (SD)	9.9 (1.0)
**Nutritional assessment**	
Haemoglobin, g/dL, mean (SD)	12.3 (2.0)
Mean cell volume, fL, mean (SD)	87.6 (6.3)
Albumin, g/L, mean (SD)	35.7 (7.9)
Urea, mmol/L, mean (SD)	4.7 (6.1)
Creatinine, μmol/L, mean (SD)	68.3 (24.4)
Potassium, mmol/L, mean (SD)	3.9 (0.4)
Sodium, mmol/L, mean (SD)	139.2 (2.6)
Magnesium, mmol/L, mean (SD)	0.82 (0.09)
Corrected calcium, mmol/L, mean (SD)	2.32 (0.18)
Phosphate, mmol/L, mean (SD)	1.05 (0.29)
Vitamin B12, pg/mL, mean (SD)	435.0 (323.5)
Folate, ng/mL, mean (SD)	8.95 (5.05)
Ferritin, ug/L, mean (SD)	187.4 (385.3)
Iron, mcg/dL, mean (SD)	9.28 (6.74)
Transferrin saturation, %, mean (SD)	17.7 (11.7)
Vitamin D, μmol/L, mean (SD)	54.1 (32.0)

Abbreviations: SD, standard deviation; HU, Hounsfield units

### Computed Tomography Assessment of Body Composition

**Figure 1.**
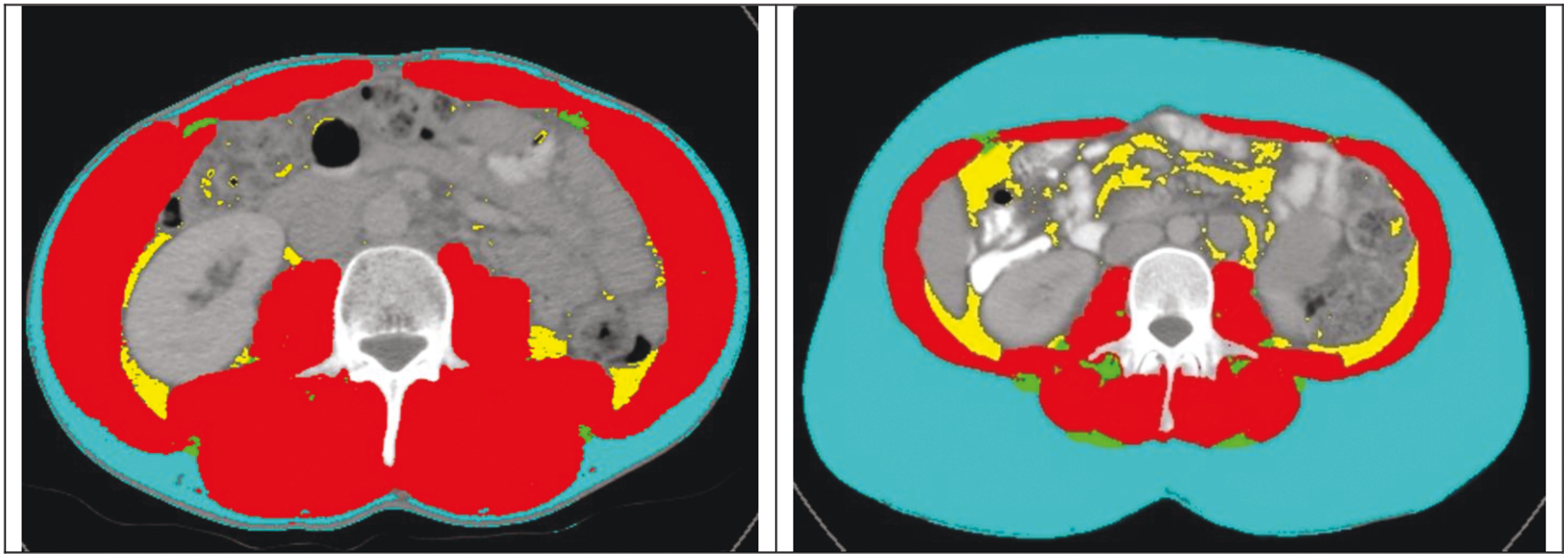
Axial CT images at the L3 level from 2 different patients with SliceOmatic (Tomovision) body composition analysis. Color overlay demonstrates subcutaneous fat area (blue), visceral fat area (yellow), intramuscular adipose tissue (green), and muscle attenuation (red) muscle (red), visceral fat (yellow), and IMAT (green).


All patients had Axial CT scans performed using a multislice Somatom Sensation scanner (Siemens Healthcare, Germany). For data analysis, preoperative CT scans were preferentially selected, with those within 6 months prior to surgery deemed eligible for study inclusion and analysis and the imaging closest to the date of surgery utilized. The 6-month inclusion window was selected for logistical reasons to capture preoperative imaging for patients undergoing both elective and emergency surgical presentations. Images at the level of the third lumbar vertebra, which displayed both transverse processes, were visible and analyzed by a single blinded investigator (J.F.) to determine the cross-sectional area (cm^2^) of each tissue compartment including psoas, paraspinal, and abdominal wall muscles using sliceOmatic 5.0 (Tomovision, Canada), applying an automated algorithm utilizing CT Hounsfield unit thresholds of –29 to 150 for skeletal muscle and –50 to –150 for adipose tissue.^34-36^ The following measures were determined in alignment with the investigative focus on sarcopenia, obesity, and myosteatosis: total fat area (TFA), subcutaneous fat area (SFA), visceral fat area (VFA), lean tissue area (LTA), intramuscular adipose tissue (IMAT, all cm^2^), and muscle attenuation (HU), as seen in Figure 1. Lean body mass (LBM) and fat mass (FM) were derived using the following formulas, which were developed and validated against DXA as standard^34,36^:

$$\begin{array}{l} LBM\;\left(kg\right)=0.30\times \left[Lean\;Tissue\;Area_{\left[L3\right]}\left(cm^{2}\right)\right]+6.06 \end{array}$$

$$\begin{array}{l} FM\;\left(kg\right)=0.042\times \left[Total\;Fat\;Area_{\left[L3\right]}\left(cm^{2}\right)\right]+11.2 \end{array}$$

Visceral obesity was defined as VFA greater than 163.8 cm^2^ for men and 80.1 cm^2^ for women.^35^

### Statistical Analysis

Data were analyzed using GraphPad Prism (v.6.0) for Windows, GraphPad software (San Diego, CA, USA) and SPSS (v.23.0) software (SPSS, Chicago, IL, USA). Univariate comparisons between groups were performed using the Student *t* or Mann-Whitney *U* tests for continuous variables or χ^2^ or Fischer exact test for categorical variables. The following factors were included in the multivariable analysis: clinicopathologic factors such as age, sex, ASA grade, biologic therapy, corticosteroid therapy, disease type (stricturing, penetrating, nonstricturing, nonpenetrating), and urgency of surgery, as well as nutritional factors such as hemoglobin, albumin, VFA, SFA, TFA, LTA, visceral obesity, IMAT, and muscle attenuation (Table 3, 4). Specific postoperative complications accounted for on multivariable analysis included reoperation, infective complication, anastomotic leak, venous thromboembolism (VTE), wound complication, wound infection, any complication, and incisional hernia (Table 5). These values were inputted into multivariable linear, logistic, or Cox proportional hazards regression models using a forward stepwise selection procedure. The urgency of surgery was accounted for in the multivariable analysis models. As such, all presented multivariable results do account for the acuity of presentation; hence all such results hold, irrespective of surgical acuity. Data are reported as mean +/- standard deviation unless otherwise specified. All statistical analyses were 2-tailed with the threshold of significance set at *P* < .05.

**Table 3.** Univariable and multivariable logistic regression analysis of body composition and postoperative morbidity.

	Univariable		Multivariable	
	*P*	OR (95% CI)	*P*	OR (95% CI)
**Clinicopathologic factors**				
Age, years	.010*	1.04 (1.01–1.06)	.465	-
Sex, male vs female	.776	1.11 (0.53–2.34)	.185	-
ASA grade, 2-3 vs 1	.269	1.58 (0.70–3.58)	.670	-
Biologic therapy	.205	0.57 (0.23–1.37)	.284	-
Corticosteroid therapy	.167	1.84 (0.77–4.37)	.452	-
Type of disease^#^	.838	-	.600	-
Urgency of surgery, urgent vs planned	.443	1.37 (0.61–3.09)	.015*	3.69 (1.29-10.58)
**Nutritional factors**				
Haemoglobin, g/dL	.115	0.86 (0.71–1.04)	.228	-
Albumin, g/L	.143	0.96 (0.92–1.01)	.451	-
Visceral fat area,	.078	1.01 (1.00–1.01)	.942	-
Subcutaneous fat area, cm^2^,	.336	1.00 (0.99–1.00)	.722	-
Total fat area, cm^2^	.141	1.00 (0.99–1.00)	.730	-
Visceral obesity	.210	1.77 (0.73–4.30)	.836	-
Lean tissue area, cm^2^	.534	1.00 (0.99–1.01)	.978	-
Intramuscular adipose tissue, cm^2^	.015*	1.07 (1.01–1.13)	.037*	1.08 (1.01-1.16)
Muscle attenuation, HU	.008*	0.95 (0.91–0.99)	.804	-

^#^Analyzed as a categorical variable, category *P* values and odds ratios not significant.

Abbreviations: OR, odds ratio; CI, confidence interval; HU, Hounsfield units.

**Table 4.** Univariable and multivariable linear regression analysis of body composition and comprehensive complications index

	Univariable		Multivariable	
	*P*	β (SE)	*P*	β (SE)
**Clinicopathologic factors**				
Age, years	.005*	0.29 (0.10)	.697	-
Sex, male vs female	.485	2.25 (3.21)	.986	-
ASA grade, 2-3 vs 1	.155	5.08 (3.55)	.084	-
Biologic therapy	.690	-1.41 (3.53)	.983	-
Corticosteroid therapy	.011*	9.09 (3.49)	.923	-
Type of disease^#^	.658	-	.967	-
Urgency of surgery, urgent vs planned	.246	4.31 (3.69)	.950	-
**Nutritional factors**				
Haemoglobin, g/dL	.115	-0.15 (0.10)	.616	-
Albumin, g/L	.002*	-0.67 (0.21)	.004*	-0.66 (0.22)
Visceral fat area,	.296	0.03 (0.02)	.546	-
Subcutaneous fat area, cm^2^,	.675	0.01 (0.01)	.843	-
Total fat area, cm^2^	.462	0.01 (0.01)	.662	-
Visceral obesity	.339	3.96 (4.12)	.668	-
Lean tissue area, cm^2^	.475	-0.04 (0.05)	.935	-
Intramuscular adipose tissue, cm^2^	<.001*	0.75 (0.20)	.029*	0.49 (0.22)
Muscle attenuation, HU	<.001*	-0.58 (0.16)	.296	

^#^Analyzed as a categorical variable, category *P* not significant. *Indicates statistically significant result.

Abbreviations: SE, standard error; HU, Hounsfield units.

**Table 5.** Multivariable analysis of the association of body composition parameters and specific postoperative complications

	Visceral Fat Area, cm^2^		Subcutaneous Fat Area, cm^2^		Total Fat Area, cm^2^		Visceral Obesity		Lean Tissue Area, cm^2^		Intramuscular Adipose Tissue, cm^2^		Muscle Attenuation, HU	
	*P*	OR (95% CI)	*P*	OR (95% CI)	*P*	OR (95% CI)	*P*	OR (95% CI)	*P*	OR (95% CI)	*P*	OR (95% CI)	*P*	OR (95% CI)
Any complication	.942	-	.722	-	.730	-	.836	-	.978	-	.037*	1.08 (1.01-1.16)	.804	-
Reoperation	.489	-	.057	-	.489	-	.653	-	.649	-	.920	-	.588	-
Infective complication	.133	-	.690	-	.370	-	.641	-	.530	-	.649	-	.632	-
Anastomotic leak	.983	-	.585	-	.684	-	.449	-	.637	-	.318	-	.188	-
Venous thromboembolism	.028*	1.02 (1.00-1.05)	.403	-	.403	-	.678	-	.479	-	.931	-	.755	-
Wound complication	.519	-	.460	-	.404	-	.140	-	.530	-	.376	-	.266	-
Wound infection	.983	-	.983	-	.042*	1.00 (1.00-1.01)	.434	-	.283	-	.545	-	.991	-
Incisional hernia	.922	-	.075	-	.922	-	.940	-	.608	-	.852	-	.878	-

Abbreviations: OR, odds ratio; CI, confidence interval; HU, Hounsfield units

## Results

### Patient Characteristics

Patient characteristics are demonstrated in Table 1. One hundred twenty-four consecutive patients that met the previously stated inclusion criteria were identified and included in the study. The mean age was 39.3 years, with a slight female preponderance. The majority of included patients were American Society of Anaesthetists (ASA) grade 1 (72.6%, *n* = 90). In keeping with the Montreal Classification of CD, the most common CD phenotype was stricturing, occurring in 46.4% (*n* = 52), followed by nonstricturing/nonpenetrating in 25.8% (*n* = 32) and penetrating disease in 16.9% (*n* = 21). Perianal disease modification as per the classification was not included. Site of disease at diagnosis was classified similarly, with the most common location of disease in the present study being ileocolonic in 50% (*n* = 62), followed by colonic in 20.2% (*n* = 25), and terminal ileum in 13.7% (*n* = 17). Extraintestinal manifestations of CD were reported in 13.7% (*n* = 17), which included arthritis (rheumatic, psoriatic, and enteropathic), uveitis, erythema nodosum, and pyoderma gangrenosum. The mean disease duration was 8.38 years. The majority of included patients had undergone prior medical therapy 76.6% (*n* = 95), with previous biologic therapy in 34.6% (*n* = 43). Previous surgery for CD was reported in 26.6% (*n* = 33). The predominant indication for surgery was stricture formation in 42 patients (33.9%), with disease refractory to medical management in 26 patients (20.9%), and the majority of patients underwent a planned surgery 77.4% (*n* = 96), with 6.5% (*n* = 8) undergoing emergency surgery. The most common procedure was an ileocolic resection, performed in 69.6% (*n* = 87). Postoperative complications with a Clavien-Dindo grade >1 occurred in 49 patients (39.5%), with a median length of stay (LOS) of 13 days, critical care LOS of 0 days, and CCI of 9.49.

### Body Composition and Nutritional Parameters

#### Adiposity

**Figure 2.**
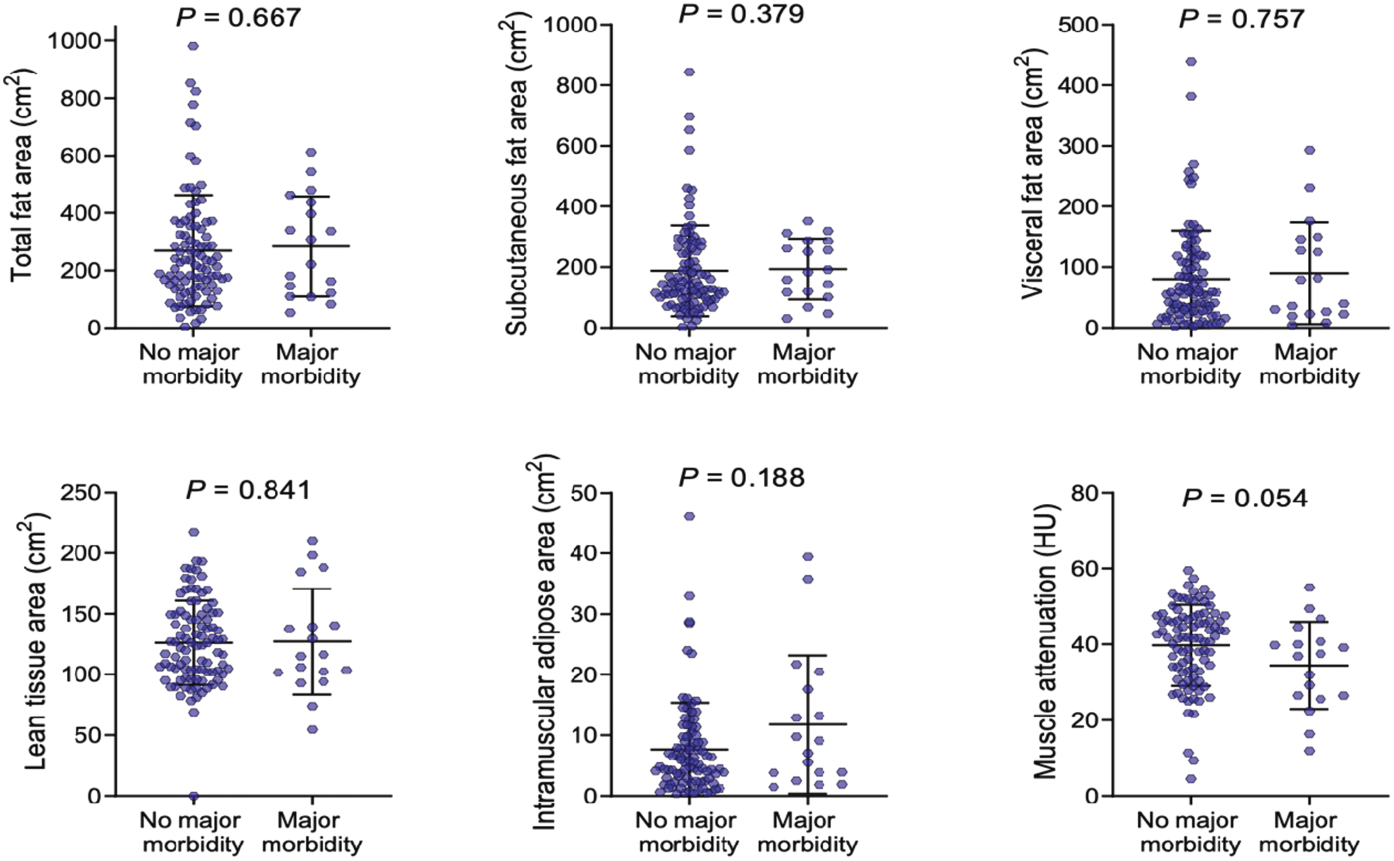
Body composition and major postoperative morbidity among patients with Crohn’s disease undergoing surgery. Mann-Whitney *U* test.

Baseline body composition and nutritional parameters are demonstrated in Table 2. Mean fat mass was 22.7 kg, with visceral obesity evident in 21.8% (*n* = 27; valid % = 23.9). Increased fat stores, as determined by fat mass (*P* = .018), TFA (*P* = .018), SFA (*P* = .030), VAT HU (*P* = .014) but not VFA (*P* = .052) were associated with an increased risk of emergency presentation. There was no observed association between markers of body composition and major post-operative moribidity among included patients (Figure 2). Visceral obesity was associated with greater age (36.4 ± 12.7 vs 51.1 ± 19.2 years, *P* < .001) and higher ASA grade (*P* < .001) but was not associated with disease phenotype. There was a trend towards increased visceral obesity (20.6% vs 38.7%, *P* = .062) and myosteatosis (10.5 ± 9.7 vs 7.1 ± 7.3, *P* = .085) among patients treated with corticosteroids preoperatively, but neither reached statistical significance. Patients with visceral obesity were less likely to present as an emergency (20.8% vs 38.5%, *P* = .021) and trended toward being less likely to present with abscess formation (7.4% vs 24.4%, *P* = .055). No significant association between markers of adiposity and biochemical nutritional parameters was observed.

#### Sarcopenia and myosteatosis

**Figure 3.**
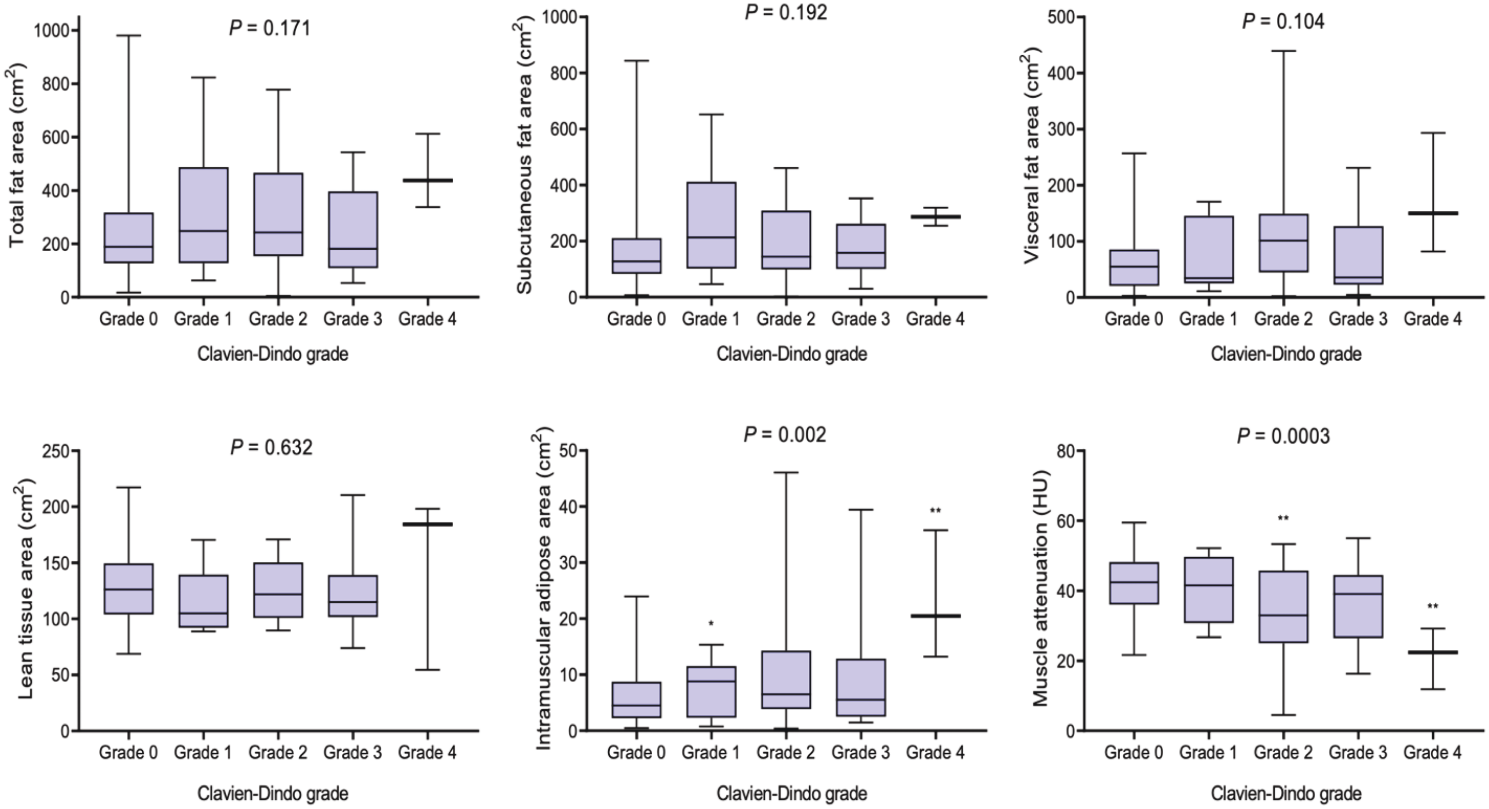
Body composition and severity of postoperative morbidity following surgery for Crohn’s disease. One-way ANOVA, Kruskall-Wallis test as appropriate.

Mean LBM was 9.9 kg. Lean body mass was significantly lower among patients who had previously undergone surgery for CD (9.5 ± 0.9 vs 10.0 ± 1.0 kg, *P* = .018), but LBM was not associated with emergency presentation (*P* = .811) or specific disease phenotype (*P* = .844). Myosteatosis markers IMAT (*P* = .002) and muscle attenuation (*P* = .0003) were associated with increased grade of complication (Figure 3). Patients treated with prior biologic therapy exhibited markers of nutritional impairment including reduced total protein (*P* = .031), magnesium (*P* = .010), and iron (*P* = .039), although patients on current biologic therapy independently exhibited markers of nutritional impairment related to folate (*P* = .037). However, body composition per the investigated parameters was similar to those without a history of biologic treatment.

Reduced LBM, indicative of sarcopenia, was associated with markers of nutritional status, with lower baseline haemoglobin (*P* < .001), haematocrit (*P* < .001), albumin (*P* = .044), and reduced protein metabolism, as indicated by urea (*P* = .017) and creatinine (*P* < .001). Similarly, reduced muscle attenuation, suggestive of myosteatosis, was associated with reduced mean cell volume (*P* = .024), total protein (*P = *.020), albumin (*P* = .004), and magnesium (*P* = .024).

### Body Composition and Postoperative Outcomes

On univariable analysis (Table 3), increasing age (odds ratio [OR], 1.04; 95% CI, 1.01-1.06; *P* = .010) and myosteatosis as determined by both increased IMAT (OR, 1.07; 95% CI, 1.01–1.13; *P* = .015), and reduced muscle attenuation (OR, 0.95; 95% CI, 0.91-0.99; *P* = .008) were associated with increased risk of postoperative morbidity. Multivariable analysis confirmed that IMAT was independently associated with increased risk of postoperative morbidity (OR, 1.08; 95% CI, 1.01–1.16; *P* = .037; Table 5). Similarly, on univariable analysis (Table 4), increasing age (*P* = .005), corticosteroid use (*P* = .011), lower serum albumin (*P* = .002), and measures of myosteatosis, IMAT (*P* < .001) and muscle attenuation (*P < *.001) were associated with increased comprehensive complications index, a marker of overall postoperative morbidity. On multivariable analysis, reduced serum albumin (*P* = .004) and increased IMAT (*P* = .029) were independently predictive of CCI. Reduced LTA (*P* = .007) and LBM (*P* = .007), indicative of sarcopenia, and myosteatosis markers, muscle attenuation (*P* = .001) and IMAT (*P* = .006), were associated with increased inpatient length of stay on univariable but not multivariable analysis.

Measures of adiposity were not associated with increased overall postoperative morbidity; however, on assessment of specific postoperative complications (Table 5), increased VFA was independently associated with postoperative venous thromboembolism (OR, 1.02; 95% CI, 1.00-1.05; *P* = .028), while TFA predicted increased risk of wound infection (OR, 1.00; 95% CI, 1.00-1.01; *P* = .042).

## Discussion

The aim of the present study was to determine the impact of sarcopenia, myosteatosis, and obesity on clinical outcomes following surgery for CD. Several previous studies have focused solely on the role of sarcopenia, highlighting the negative impact of sarcopenia on IBD patients postoperatively.^27,37^ Methods for measurement of sarcopenia have been reexamined in recent years, and the present study harnessed modern imaging techniques with low-dose CT to accurately assess preoperative body composition in CD patients. In addition to sarcopenia, measurements of obesity and myosteatosis were included to provide a more comprehensive classification of preoperative body composition status and therefore a more accurate assessment of the impact of these parameters on clinical outcomes. The main findings were that while both sarcopenia and myosteatosis were associated with impaired baseline nutritional markers, only the myosteatosis markers, IMAT, and muscle attenuation were associated with the rate and complexity of postoperative morbidity. Interestingly, while measures of adiposity were not associated with overall postoperative morbidity, increased VFA independently predicted the specific morbidities of venous thromboembolism and surgical site infections. This suggests that increased adiposity may reflect preservation of nutritional status and relatively more quiescent disease phenotype; and despite specific increased perioperative risks, overall morbidity is not independently impacted. However, impaired muscle mass and function represents an important modifiable risk factor for patient optimization to improve outcomes in the surgical management of CD.

In contrast to much of the literature,^37,38^ sarcopenia as determined by reduced LBM, was not significantly associated with worse postoperative outcomes. While sarcopenia has been extensively evaluated in cancer, in the setting of IBD there is a wide range of variability in its reporting due to a multitude of factors: heterogeneity of the patient cohort, wide age distribution, chronic inflammatory nature of disease, chronic malnutrition, immunosuppression, and lack of standardized methods of measurement. The prevalence of sarcopenia in patients with IBD, defined by low skeletal muscle mass, has been reported as high as 60% in patients with CD as measured on the DEXA scan.^39^ The mechanism by which sarcopenia may effect postoperative outcomes is based on a patient’s physiological reserve and their ability to recover in the early postoperative setting.^14-16^ While Huang et al^40^ found a clinically significant impact of sarcopenia on postoperative complications in colorectal cancer, a recent paper by O’ Brien et al found no such correlation.^41^ It could also be hypothesized that due to the MDT approach to IBD at our institution, preoperative patient optimization, elective surgery in the majority of patients, and ERAS protocols, the previously observed postoperative complications may have been mitigated.^42^

Unlike sarcopenia, myosteatosis as determined by increased IMAT and reduced muscle attenuation was independently predictive of increased overall morbidity and CCI on multivariable analysis. Unsurprisingly, myosteatosis was significantly associated with markers of impaired nutritional status at presentation, suggesting that it may reflect disease-associated malnutrition and muscle wasting among patients with CD. The mechanisms linking myosteatosis and adverse postoperative outcomes are currently incompletely understood. Several hypotheses exist, the most prominent focusing on the relationship between myosteatosis, systemic inflammation, and cachexia. Cachexia is a multifactorial syndrome characterized by pathological muscle wasting^43^ and may be associated with myosteatosis, which may also be characterized by an inflammatory catabolic state.^44-46^ Myosteatosis is also linked with increased insulin resistance through the impairment of nutritive blood flow to muscle fibers and the induction of local metabolic changes due to the secretion of adipokines; it has also been implicated as having increased prevalence amongst patients with increased visceral adiposity.^45,47,48^ Increased markers of systemic inflammation and hypoalbuminemia have been associated with increased myosteatosis in colorectal cancer.^49^ Sustained activation of a systemic inflammatory response is a significant contributor to the development of cachexia, as evidenced by raised levels of pro-inflammatory cytokines IL-1, IL-6, and TNF-α in these patients.^50^ Myosteatosis and cachexia may manifest clinically in the frailty phenotype, a distinctive health state characterized by reduced physiologic reserve.^51,52^ Recent data indicate that myosteatosis is a stronger predictor of increased frailty among older patients than sarcopenia alone,^53^ which may underlie the finding of adverse outcomes among patients with CD in the present study.

It has been established that food avoidance due to negative postprandial visceral symptoms is common among CD patients.^54^ Although, the methods by which patients with CD avoid certain food groups to avoid disease flare are incompletely understood, though it has been reported that patients may avoid vegetables, dairy products, and fats. In contrast to this, it has also been reported that while undergoing a disease flare, CD sufferers may avoid proteins and fats, essential for adequate protein metabolism.^55-57^ These 2 behaviors in tandem could be hypothesized to exacerbate the preponderance of these patients towards a chronic malabsorptive state, resulting in a clinical diagnosis of sarcopenia and myosteatosis.^58^ Casanova et al found that patients with IBD and clinical malabsorption had reduced hand-grip strength, a significant marker of muscle quality and one of the foundations of a diagnosis of sarcopenia compared with well-nourished patients.^10,57^

Obesity was observed in 21% of patients in the current study, a similar percentage observed in other studies of patients with CD.^59^ While our study showed there was a reduced risk of emergency presentation in obese patients, there was an observed association with increased ASA grade and older age. Crohn’s disease patients, despite having a chronic inflammatory disease and being at risk of chronic malnutrition as well as chronic muscle atrophy, have been noted to have an increasing prevalence of obesity.^60,61^ While the current study did not assess obesity as a lone entity in identifying risk of postoperative complications, overall body composition parameters were investigated to more accurately identify preoperative risk factors for adverse surgical outcomes in CD patients. It is accepted that BMI alone is not accurate enough to determine body composition in the setting of IBD, and many patients may fall within normal parameters.^23^ Given that almost all CD patients undergo cross-sectional imaging in diagnostic workup in an Irish setting, we sought to identify parameters identifiable on CT imaging which may confer negative outcomes. The prevalence of obesity has also been observed to be higher amongst CD patients with mild or well-controlled disease, suggesting disease quiescence may contribute to this phenomenon, perhaps putting them at further risk of societal risks such as sedentary lifestyle and poor diet associated with the endemic obesity observed in Western society.^61^ This finding has further added to the theory of obesity being a form of malnutrition in CD, with sarcopenic obesity being observed in 20% of CD patients in 1 study.^59^ The current literature is not in agreement on the effect of obesity on surgical outcomes in CD patients,^62^ with several studies observing an increased postoperative risk and worse surgical outcomes,^59^ while others suggest a more quiescent disease process with no observed negative impact on postoperative outcomes.^63^ Notably, although measures of adiposity were associated with increased specific morbidity in our study, no increase in overall morbidity was observed in this group, suggesting that these issues may be managed conservatively without impacting overall recovery, critical care utilization, or length of stay.

Given the association between myosteatosis and adverse outcomes in our study, a key avenue for future research is to identify strategies to optimize body composition with the view to improve operative outcomes. In this regard, prehabilitation and rehabilitation protocols have significant appeal in the management of patients with CD. Increased physical activity is associated with retention of LBM among patients with IBD and increased cardiopulmonary fitness among patients undergoing rehabilitation following treatment for other complex attritional diseases.^64^ Further understanding of the aetiology of myosteatosis in autoimmune pro-inflammatory states such as IBD and its potential for targeted treatment or reversibility with preoperative optimization prior to surgery may provide more favorable patient outcomes. Furthermore, the role of anti-inflammatory treatments in the treatment of sarcopenia and cachexia is a topic of much current interest.^59^ Immunonutrition involves the addition of omega-3 polyunsaturated fatty acids (PUFA), arginine, and glutamine to nutritionally complete regimens. These formulations modulate the production of pro-inflammatory mediators through the arachidonic acid pathway and represent a potential treatment avenue for patients with myosteatosis, having shown promising results in other fields.^65,66^

We acknowledge a number of limitations. Firstly, although this study included consecutively treated patients, it may be underpowered to detect smaller effects with respect to operative outcomes compared with a multicenter analysis. This is particularly pertinent given the heterogeneity of presentations typical in a CD cohort, which fundamentally limits subgroup analysis. Secondly, further study is needed to determine the impact of preoperative and nonoperative treatment strategies, particularly biologic agents, on skeletal muscle metabolism. A further limitation to note is an inherent spectrum bias by the sole inclusion of CD patients undergoing resectional surgery, fundamentally limiting the generalizability of interpretation of results to the broader CD patient cohort. Another limitation is the duration of time between CT imaging and surgical intervention. It is not known if the markers of body composition are stable over this time in patients with Crohn’s disease; the present analysis shows that some of these markers are associated with disease severity and hence may change over time with disease activity and with treatment.^67^ The etiology of the findings in this study may be less clear due to its retrospective design, which limits the ability to establish causality with the findings. The measures of body composition did not include a skeletal muscle index (SMI), which may be used for diagnosis of sarcopenia via CT scan, nor did it include BMI; this was due to the inability to correct for height given the retrospective nature of the study. However, while indicators of sarcopenia including LBM and measurement of SFA at the level of L3 were analyzed, it is important to note that interpretation of muscle parameters is difficult, as healthy population cutoffs are not well defined. Furthermore, the impact of age, sex, BMI, and ethnicity all play a role in their interpretation, respectively.^67-70^ The use of complications indices including the Clavien-Dindo classification has several drawbacks; while widely accepted in the field of general surgery, it must be noted that its interpretation may be limited in certain circumstances.^71^ The authors have endeavoured to avoid this by also availing of the CCI, which has been reported as being a more sensitive complication index in colonic surgery.^72^ A prospective cohort study would help to alleviate inherent selection bias, variance in timing of surgical intervention, imaging assessment, and biochemical assessments, which would offer meaningful insights into the impact of body composition on the postoperative course and outcomes in CD patients. Furthermore, a formal nutritional assessment questionnaire with pre- and postoperative quality of life data could be informative. Previous studies have shown that IBD patients who are hospitalized frequently have a higher protein-calorie deficit when compared with nonIBD patients.^73^ Furthermore, the inclusion of emergency and nonemergent cases may confound certain results due to the known increased morbidity of complications in CD patients undergoing emergency procedures.^74,75^

In conclusion, the present study found that myosteatosis was independently associated with increased postoperative morbidity in patients undergoing resectional surgery for CD. The role of sarcopenia in the present study concurs with findings of more recent studies that suggest it may have a lesser role in patient outcomes in IBD surgery; however, it is still prevalent within this patient cohort and may offer useful insight into the overall preoperative body composition of patients at potential at risk from chronic inflammation and malabsorption. In terms of myosteatosis, preoperative measures to optimize muscle mass and function, and targeting the systemic inflammatory response to minimize catabolism, have appeal in the evolving goal to personalize the approach to both operative and nonoperative care for patients with CD.

## Data Availability

The data sets used and/or analyzed in this study are available from the authors upon reasonable request.

## Abbreviations:

ASA: American Society of Anaesthesiologists
CI: confidence interval
CCI: comprehensive complications index
CD: Crohn’s disease
DXA: dual energy X-ray absorptiometry
IBD: inflammatory bowel disease
IMAT: intramuscular adipose tissue
LBM: lean body mass
LOS: length of stay
LTA: lean tissue area
MDT: multidisciplinary team
MRE: magnetic resonance enterogram
MUST: malnutrition universal screening tool
SFA: subcutaneous fat area
SMI: skeletal muscle index
UC: ulcerative colitis
VFA: visceral fat area
VTE: venous thromboembolism
